# Candidate Genes and Quantitative Trait Loci for Grain Yield and Seed Size in Durum Wheat

**DOI:** 10.3390/plants10020312

**Published:** 2021-02-05

**Authors:** Giacomo Mangini, Antonio Blanco, Domenica Nigro, Massimo Antonio Signorile, Rosanna Simeone

**Affiliations:** 1 Institute of Biosciences and Bioresources, National Research Council, Via Amendola 165/A, 70126 Bari, Italy; 2 Department of Soil, Plant and Food Sciences, Genetics and Plant Breeding Section, University of Bari Aldo Moro, Via Amendola 165/A, 70126 Bari, Italy; antonio.blanco@uniba.it (A.B.); domenica.nigro@uniba.it (D.N.); massimoantonio.signorile@uniba.it (M.A.S.); rosanna.simeone@uniba.it (R.S.)

**Keywords:** wheat, grain yield, grain size, candidate genes, QTL cluster

## Abstract

Grain yield (YLD) is affected by thousand kernel weight (TKW) which reflects the combination of grain length (GL), grain width (GW) and grain area (AREA). Grain weight is also influenced by heading time (HT) and plant height (PH). To detect candidate genes and quantitative trait loci (QTL) of yield components, a durum wheat recombinant inbred line (RIL) population was evaluated in three field trials. The RIL was genotyped with a 90K single nucleotide polymorphism (SNP) array and a high-density genetic linkage map with 5134 markers was obtained. A total of 30 QTL were detected including 23 QTL grouped in clusters on 1B, 2A, 3A, 4B and 6B chromosomes. A QTL cluster on 2A chromosome included a major QTL for HT co-located with QTL for YLD, TKW, GL, GW and AREA, respectively. The photoperiod sensitivity (*Ppd-A1*) gene was found in the physical position of this cluster. Serine carboxypeptidase, Big grain 1 and β-fructofuranosidase candidate genes were mapped in clusters containing QTL for seed size. This study showed that yield components and phenological traits had higher inheritances than grain yield, allowing an accurate QTL cluster detection. This was a requisite to physically map QTL on durum genome and to identify candidate genes affecting grain yield.

## 1. Introduction

Durum wheat (*Triticum turgidum* L. ssp. *durum*) is grown on about 17 million hectares of land worldwide [1]. The Mediterranean Basin is the main durum wheat producing area, as well as its most significant import market and its largest consumer, being durum wheat primarily used for pasta, couscous, bulgur and farik production. The world’s projected population growth will result in a higher production demand, thus increasing grain yield per unit area will be of great importance to face this mounting challenge [2]. The rate of wheat production has been increasing by 0.9% per year, which is much less than the required 1.8%, suggesting that the improvement of wheat yield must be further increased [3]. Grain yield is conventionally expressed as the product of a different number of sub-traits named “yield components”, covering two main parameters: number of spikes per area and grain yield per spike. Grain yield per spike comprises grain number per spike and grain weight, usually expressed as thousand kernel weight (TKW). Grain weight is mainly and tightly underpinned by grain morphology including grain area, grain length and grain width. In the domestication process and breeding history, grain size has been a major selection and breeding target, thus being widely selected and manipulated to increase grain yield [4].

Understanding the genetic and molecular determinants of grain size might provide valuable information on the markers to be used for improving grain yield. As extensively reviewed by Nadolska-Orczyk et al. [5], the major genes determining yield-related traits can be classified in several groups. Among these, there are: transcription factors, which can affect grain number by regulating spike development; genes involved in metabolism or signaling of growth regulators, affecting plant architecture; genes determining cell division and proliferation, related to grain size; floral regulators, which regulate inflorescence architecture and seed number; and genes involved in carbohydrate metabolism, affecting plant architecture and grain yield.

The most advanced knowledge on the genetic factors controlling grain size were detected in rice. Indeed, quantitative genetic studies and map-based cloning have allowed several genes associated with grain size and weight in rice to be isolated [6]. *OsGW2* was found to affect both grain weight and width [7], as well as its wheat orthologue, *TaGW2*, which was mapped on chromosome 6A [8] and found to be related to grain weight, grain width and grain length [9]. Similarly, allelic variation at *TaGS-D1*, the wheat orthologue of rice *GS3* [10], showed main effects on grain weight and kernel size [11]. Genes involved in starch and sucrose metabolism pathways were also shown to affect grain weight, such as *TaSus1* and *TaSus2* [12,13] and *TaCwi-A1* [14]. In addition, major phenology loci can exhibit pleiotropic effects on spike and kernel traits. These include the locus *Rht1*, which have been intensively used since the Green Revolution in breeding programs worldwide, and the major photoperiod sensitivity locus *Ppd1*, which is known to affect heading time as well as a range of other traits [15]. *Ppd1* genes play an important role in wheat growth and development regulation by affecting the accumulation and distribution of dry matter within the plant and modifying source-sink equilibrium [16,17]. Indeed, it has been estimated that up to 35% of wheat grain yield increment observed in Europe can be attributed to photoperiod insensitivity [18].

In the last decade, single nucleotide polymorphism (SNP) markers have become fundamental for both genetic studies and breeding programs, and the development of wheat high-density SNP array [19] provided the most innovative tool for candidate genes searching by quantitative trait loci (QTL) mapping or genome wide association (GWAS) studies. Moreover, the recently released common and durum wheat genome could provide valuable information to decipher complex marker-traits associations and simplify the searching and discovery of genes underlying important agronomic traits [20].

In the present study, a high-density genetic linkage map based on iSelect 90K SNP markers was developed using the durum wheat Liberdur x Anco Marzio recombinant inbred line (RIL) population that was evaluated to detect QTL controlling grain yield components and grain size. Furthermore, the availability of a full reference durum wheat genome [21], allowed the physical projection of the identified QTL genetic intervals on the reference Svevo genome, as well as the detection of candidate genes involved in the phenotypic control of grain yield and grain size.

## 2. Results

### 2.1. Phenotypic Characterization

The two parental lines, Liberdur and Anco Marzio, and the RILs mapping population were evaluated for yield components and grain size traits (grain yield (YLD), plant height (PH), heading time (HT), thousand kernel weight (TKW), grain length (GL), grain width (GW) and grain area (AREA)) for three growing seasons (2016, 2017 and 2018) in southern Italy (Valenzano, Bari, Italy). Analysis of variance revealed highly significant differences among RILs for all traits in each year, while the combined analysis across years revealed significant effects of RILs, years and genotype × year interaction (Table 1 and Table S1). However, although the strong season effect, the genotype variability was higher than genotype × year component for all traits apart from YLD.

Mean values of Liberdur, Anco Marzio, and RILs in single season and across the three field trials are reported in Table 2. The two parents showed significant differences for HT, PH, AREA in all three seasons, and for TKW, GL and GW in two trials. In particular, Anco Marzio has generally larger grains compared to those of Liberdur.

**Figure 1:**
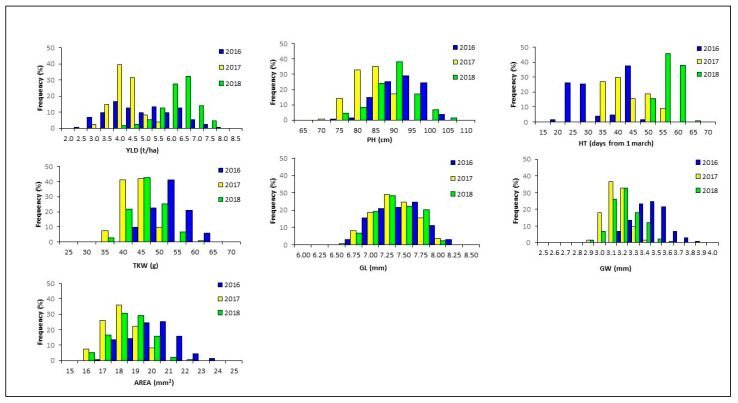
Frequency distribution of grain yield (YLD), plant height (PH), heading time (HT), thousand kernel weight (TKW), grain length (GL), grain width (GW), grain area (AREA) in the Liberdur x Anco Marzio RILs mapping population evaluated at Valenzano (Bari, Italy) for three years.

Analysis of the frequency distribution of traits in the RIL population was performed to have a preliminary idea of the genetic basis for each trait. A bimodal distribution was observed for HT indicating a single locus segregating in the RIL mapping population (Figure 1). The normal distribution obtained for YLD, PH, TKW and grain size parameters indicated that several loci, each contributing to a small proportion of the total variation, control the examined traits. High transgressive segregation was recorded for all traits suggesting the presence of superior alleles for grain yield components in both parental lines. Low values of broad sense heritability were estimated for YLD, confirming that the trait was strongly affected by environmental conditions and genotype × year interaction. High heritability values, exceeding 0.55, were found for HT, GL and AREA while TKW heritability values ranged from 0.45 to 0.72 (Table 2).

Phenotypic correlation among the traits in each year and across the three years are reported in Table 3. As expected, GL, GW and AREA were inherently correlated in each year and across years. TKW showed a high positive correlation with grain size traits.

### 2.2. Genetic Linkage Map

Out of 81,587 SNP markers assayed, 5,543 (6.8%) resulted as failed and 67,999 (83.3%) were monomorphic in the mapping population. The remaining 8045 (9.86%) markers were polymorphic; however, 2686 that had more than 10% missing data and 225 with distorted segregation (at *p* ≥ 0.05 value) were excluded from further analysis. Hence, 5134 (6.29%) markers were used for the genetic map construction. The 5134 loci were grouped in 21 linkage groups when using a LOD score 5. Linkage groups were assigned to the A and B genome chromosomes using the durum consensus map [22]. Eight chromosomes (1B, 2A, 3B, 4B, 6A, 6B, 7A and 7B) were assembled in a single linkage group (Table 4). Twenty-tree loci assembled in a linkage group of 4A chromosome resulted to be coincident in the same position and therefore discarded from the QTL analysis. A total of 2085 markers were localized on the A genome with a total length of 1145.3 cM, whereas 3049 were mapped on the B genome (total length 1062.7 cM). The entire map covered 2207.9 cM with an average chromosome length of 157.7 cM. The lengths of individual chromosomes varied from 73.3 cM (chromosome 5B) to 197.2 cM (chromosome 7A). The SNP markers were generally well distributed throughout the genome, although some chromosomes exhibited higher densities. The overall SNP density was 2.3 markers/cM, with a maximum of 3.8 for chromosome 1B and a minimum of 1.2 for chromosome 4A.

### 2.3. Quantitative Trait Loci (QTL) Detection

**Figure 2:**
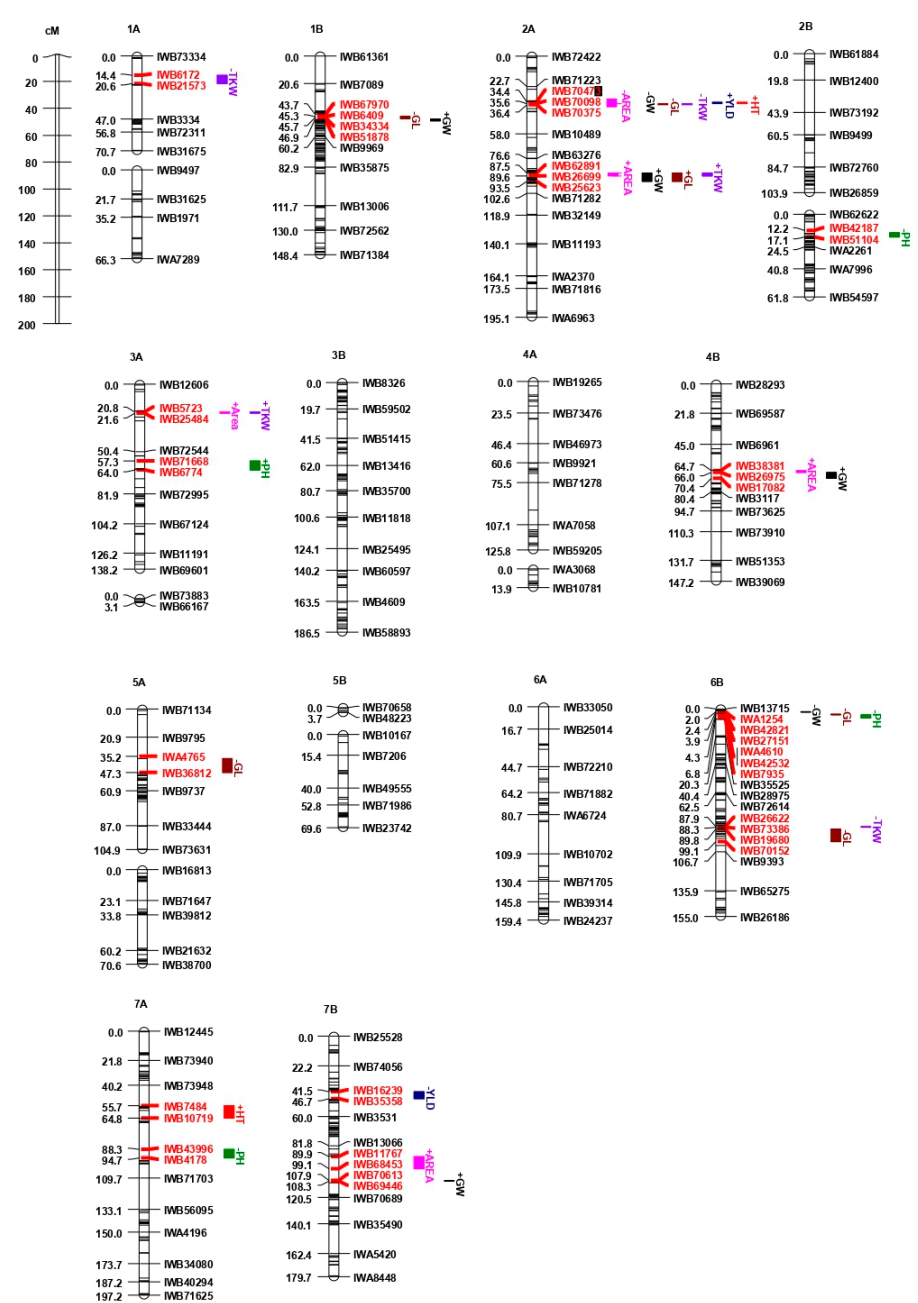
Schematic representation of the durum linkage map Liberdur x Anco Marzio with map positions of QTL for grain yield (YLD), plant height (PH), heading time (HT), thousand kernel weight (TKW), grain length (GL), grain width (GW), grain area (AREA). Each chromosome map is represented by the first and the last SNP marker, and by a SNP marker every about 20 cM. Markers are indicated on the right side and cM distances on the left side of the bar. QTL are represented by bars on the right of each chromosome bar. The + or − preceding the QTL name indicate the positive or negative additive effect of the parental line Liberdur. The left and right SNP markers of the QTL interval are indicated in red.

A total of 30 putative QTL were detected on 10 chromosomes in the durum wheat Liberdur x Anco Marzio mapping population (Table 5). Two QTL for YLD were detected on chromosomes 2A in 2016 and on chromosome 7B in 2018, each explaining 67.9% and 11.9% of the phenotypic variance, respectively. *QYLD.mgb-2A*, showed a positive additive effect provided by the parental line Liberdur (Figure 2), while *QYLD.mgb-7B* displayed the positive additive effect conferred by Anco Marzio. The higher GxA interaction variance than genotype effect prevented the QTL analysis for YLD across years.

Two QTL for HT were detected on chromosomes 2A and 7A, accounting for 2.4 to 85.7% of the explained phenotypic variance (R^2^). The −log10(P) scores of *QHT.mgb-2A* ranged from 44.6 to 69.7 and explained over 70% of the phenotypic variance in each year. This locus was stably detected in three years and across years, resulting as a major QTL, with additive effect contributed by Liberdur.

Four QTL were found to be significantly associated with PH on chromosomes 2B, 3A, 6B and 7A, accounting for 7.8% to 17.5% of the phenotypic variance. *QPH.mgb-3A* and *QPH.mgb-6B* were stably detected in two field trials and across years. The additive effects at QPH.mgb-3A and *QPH.mgb-6B* were provided by Liberdur and Anco Marzio, respectively.

Five TKW QTL were detected on chromosomes 1A, 2A, 3A and 6B, which accounted for 2.9% to 35.8% of the phenotypic variance. Of them, two stable QTL were mapped on chromosome 2A, delimited by genetic intervals 35.6–36.4 cM (*QTKW.mgb-2A.1*) and 87.5–89.6 cM (*QTKW.mbg-2A.2*), respectively. *QTKW.mbg-2A.1* and *QTKW.mbg-2A.2* additive effects were conferred by Anco Marzio and Liberdur, respectively.

Six QTL were found to be significantly associated with GL on 1B, 2A (2 QTL), 5A and 6B (2 QTL). Among them, QGL.mgb-2A.2 was declared in all years accounting for 39.1–53.8% of the phenotypic variance, thus resulting as a stable QTL. Additive effect at this locus derived from Liberdur. The *QGL.mgb-1B* and *QGL.mgb-5A* were declared in two years (both 2016 and 2018), while the remaining three QTL (*QGL.mgb-2A.1*, *QGL.mgb-6B.1* and *QGL.mgb-6B.2*) were detected in a single year. For these five QTL, the alleles for longer grain were carried by the parental line Anco Marzio.

Six QTL were found to be significantly associated with GW on 1B, 2A (2 QTL), 4B, 6B and 7B chromosomes. The *QGW.mgb-1B* and *QGW.mgb-2A.2* were stably detected and accounted for 8.4–16.3% of phenotypic variance. Both QTL showed the additive effect of Liberdur allele. The *QGW.mgb-2A.1* explained a large phenotypic variation (42%), although it was detected in a single year and across years.

Five QTL were identified for AREA on 2A (2 QTL), 3A, 4B and 7B. The *QAREA.mgb-2A.2* was found in all years and across years accounting for 25.8–47.8% of the phenotypic variance, resulting to be a stable QTL. *QAREA.mgb-4B* was found in two years (2017 and 2018) while the other three QTL (*QAREA.mgb-2A.1*, *QAREA.mgb-3A* and *QAREA.mgb-7B*) were identified in a single year. Liberdur carried the allele for larger area for all QTL, except for *QAREA.mgb-2A.1*.

Out of the 30 detected QTL, 10 were co-located in two different regions of chromosome 2A (Figure 2). The first region included six QTL (*QYLD.mgb-2A*, *QHT.mgb-2A*, *QTKW.mgb-2A.1*, *QGL.mgb-2A.1, QGW.mgb-2A.1* and *QAREA.mgb-2A.1*) in the genetic interval between 34.4 and 36.4 cM, while the second comprised four stable QTL (*QTKW.mgb-2A.2*, *QGL.mgb-2A.2*, *QGW.mgb-2A.2* and *QAREA.mgb-2A.2*) in the interval between 87.5 and 89.6 cM. Interestingly, among the five QTL for TKW, two co-located together with QTL for GL, GW and AREA on chromosome 2A (both regions), and two co-located with QTL for GL or AREA on chromosomes 3A and 6B, respectively. Four out of 6 QTL for GL co-located with QTL for GW on chromosomes 1B, 2A (two QTL) and 6B. QTL for AREA always co-located with grain traits QTL (TKW, GL, and GW) on chromosomes 2A (2 QTL), 3A, 4B and 7B.

### 2.4. Candidate Genes Involved in Grain Yield-Related Traits

To identify candidate genes in the physical regions underlying the detected QTL, each region was projected on the recently released reference durum wheat genome of cv. Svevo [21]. Regions with two or more co-locating QTL were considered as a QTL cluster (Table 6), and the sequences of flanking markers of each of them were anchored to their physical position on the durum genome. A total of eight QTL clusters were detected with size ranging from 0.4 Mbp (on chromosome 3A, where two QTL for TKW and AREA co-located) to 57.1 Mbp (on chromosome 6B, with a QTL for TKW and a GL co-locating). In the QTL clusters, 1772 annotated high-confidence (HC) genes were found, ranging from 12 (on cluster 4) to 605 (on cluster 6). Among them, only those which annotations indicated a possible involvement in seed development were retrieved [5,23]. Table 6 reports the most likely candidate genes found in each QTL cluster, based on their potential involvement in grain yield.

Among the several candidate genes localized within the QTL cluster regions, four genes were particularly noteworthy, as previously reported to be directly involved in grain yield: *TRITD2Av1G019250*, encoding a Pseudo-response regulator (*Ppd-A1*) [24] in the 2A cluster that included *QHT.mgb-2A* (Table 5); *TRITD4Bv1G171270*, encoding a Big grain 1 protein [25] in the 4B cluster, and two candidate genes encoding an acid β-fructofuranosidase (*TRITD6Bv1G005370* and *TRITD6Bv1G005450*), paralogues of the cell wall invertase gene [26], both in the 6B cluster containing QTL for GL, GW and PH.

Specific protein classes were frequently observed, such as proteins involved in ubiquitination processes, including E3 ubiquitin-protein ligase and RING U-box superfamily proteins (identified in six QTL clusters), cytochrome P450 (identified in four QTL clusters) and thioredoxins (identified in two QTL clusters), as well as serine carboxypeptidase proteins (identified in two QTL clusters).

Interestingly, in all clusters in which a QTL for AREA was detected, genes involved in auxin metabolism were found, strengthening their chances of being considered candidates for this trait: *TRITD1Bv1G118820*, *TRITD2Av1G189400*, *TRITD7Bv1G173200*, encoding protein involved in auxin response; *TRITD4Bv1G175480*, involved in auxin signaling, and *TRITD3Av1G012070*, a paralog gene of the YUC family encoding a flavin-containing monooxygenase directly involved in auxin biosynthesis. Similarly, except for one cluster on the 2A chromosome, in all QTL clusters in which the QTL for GW were co-located, genes encoding cytochrome P450 monooxygenases (*CYPs*) were found, suggesting this family as candidate for GW. The *CYPs* are a superfamily involved in several plant metabolism including biosynthesis of hormones, cell wall components, and defence compounds [27]. Recently, the *TaCYP78A3* and *TaCYP72A* genes were found to affect seed size showing the role of these genes in seed development [28,29].

## 3. Discussion

Grain yield is a typical quantitative trait controlled by a complex genetic system and strongly influenced by both environmental factors and agronomic management. Grain yield reflects the combination of kernel weight and grain number per area. Besides being one of the key components of grain yield, thousand kernel weight is often used as standard parameter for flour-milling yield and marketing standard. Thousand kernel weight is mainly and tightly underpinned by grain morphology including grain length, grain width and grain area. Grain yield can be maximized by growing varieties whose heading time allows the crop to avoid stresses during the grain-filling phase [17]. Indeed, heading time is a critical stage delimiting the duration of spike formation and marking the transition into the grain-filling period, during which grain per spike and grain weight are both defined. In the last century, grain yield improvement was obtained through increased harvest index and straw strength with the introduction of major genes conferring reduced plant height [1,5,30].

In this study, YLD, TKW, morphology grain traits (GL, GW and AREA), HT and PH were investigated by using an RILs mapping population consisting of 133 lines derived by crossing the elite durum wheat cultivars Liberdur and Anco Marzio. Plant materials were evaluated in field trials for three years.

A total of 30 QTL were identified and localized on 10 out of 14 durum wheat chromosomes. Among them, nine stable QTL for TKW (2 QTL), GL, GW (2 QTL), AREA, HT and PH (2 QTL), distributed on 1B, 2A, 3A and 6B chromosomes, detected at −log10(P) > 3.0 in at least two years and across years (Table 5). Interestingly, 6 out of 9 stable QTL were co-located in QTL clusters on chromosome 2A, while the other three were detected on 1B, 3A and 6B chromosomes (Figure 2). Furthermore, 4 out of 8 QTL clusters were mapped on 2A and 6B chromosomes, and the remaining ones on 1B, 3A, 4B and 7B.

The first QTL cluster on chromosome 2A, delimited by genetic interval 35.6–36.4 cM, included two stable QTL (*QHT.mgb-2A* and *QTKW.mgb-2A.1*) along with the QTL for YLD, GL, GW and AREA detected in a single year (2016). In this region, *QHT.mgb-2A* can be considered a major QTL involved in the phenotypic control of HT as it was declared in each field trial with a −log10(P) ranging from 44.0 to 69.7, and a R^2^ from 74.3 to 85.7, respectively. The candidate genes analysis detected a pseudo-response regulator protein (*TRITD2Av1G019250*), corresponding to a photoperiod sensitivity gene (*Ppd-A1*), within the physical position of this QTL. Wilhelm et al. [24] found two large deletions within the *Ppd-A1* gene in durum wheat (1027 and 1117 bp deletion designated as alleles ‘GS-100’ and ‘GS-105’, respectively), which remove a common region from the wild-type sequence. Our results suggested that the parental lines Liberdur and Anco Marzio could be different for the alleles at *Ppd-A1* locus. Similar conclusions were reported by Maccaferri et al. [31], who identified a QTL reducing heading date associated with *Ppd-A1* in a RILs population derived from the cross ‘Kofa’ (‘GS-100’ allele) × ‘Svevo’ (‘GS-105’ allele), thus indicating that these alleles decrease photoperiod sensitivity at different degrees. The QTL cluster with the *Ppd-A1* gene included the QTL for YLD and grain size detected in 2016. The first half of April 2016 was characterized by a severe temperature decrease compromising grain filling of early spiked RILs while later ones escaped cold damage resulting in more grain yield but with small grain. In fact, HT was significantly positively correlated with YLD (r = 0.85) and negatively with grain size traits in 2016 (*r* values ranging from −0.70 to −0.28). In addition, the QTL for HT (*QHT.mgb-2A*) and YLD (*QYLD.mgb-2A*) were associated to significant QTL with opposite effects on TKW (*QTkw.mgb-2A.1*, *QTKW.mgb-2A.1*) and grain size (*QGL.mbg-2A.1*, *QGW.mgb-2A.1*, *QAREA.mgb-2A.1*). These results suggested that *Ppd-A1* had negative pleiotropic effects on TKW and grain size, but positively affected others yield components such as grain number per unit area [32].

The second QTL cluster on chromosome 2A included four stable QTL (*QTKW.mgb-2A.2*, *QGL.mgb-2A.2*, *QGW.mgb-2A.2*, *QAREA.mgb-2A.2*) declared in three years and across years. The positive additive effect was provided by the parental line Liberdur for all four QTL. As shown by correlation data, TKW was always positively and significantly correlated to GL, GW and AREA. These results suggested that TKW improvement could be due to the grain size increase. A QTL for TKW was previously detected in this region by a genome-wide association study on a tetraploid wheat collection [33], thus validating the presence of a metaQTL regardless of the identification approach. Six putative candidate genes were included in this QTL cluster and, among them, a serine carboxypeptidase protein. Previous studies have shown that serine carboxypeptidase enzymes are involved in several biological processes, including development of plant organs [34,35], cell division [36] and cell elongation [37]. Indeed, by promoting cell division, these enzymes determine larger grain size due to an increased cell number [36]. Our result was not unexpected considering that other studies on dissecting grain size also detected a strong relation with serine carboxypeptidase proteins [38,39].

A QTL cluster on 6B chromosome included the stable QTL *QPH.mgb-6B*, which was detected in the same region where QTL for PH were already reported by Canè et al. [40] and Soriano et al. [41]. Two additional QTL co-localized in this cluster, *QGL.mgb-6B.1 and QGW.mgb-6B*, where Canè et al. [40] detected a QTL for test weight, a trait which is known to be affected by grain size [42]. Therefore, it could be supposed that high values of test weight might be determined by increasing GL and GW. The co-localization of QTL affecting different traits such as PH, GL and GW, implies closely linked genes involved in different biological processes related to yield [43]. In this QTL cluster, two candidate genes (*TRITD6Bv1G005370* and *TRITD6Bv1G005450*) encoding the acid β-fructofuranosidase enzyme were found. These two genes have 22 paralogues in the B genome of wheat, which probably diverged from a common ancestral gene and evolved by duplication. More interesting is the fact that these genes are paralogues of cell wall invertase genes. β-fructofuranosidase (EC 3.2.1.26) is indeed commonly known as invertase in plants. These genes are involved in the carbohydrate metabolic process and have a common molecular function, as invertase/fructofuranosidase belong to the glycoside hydrolase family 32 [26]. Ma et al. [14] reported an association between a cell wall invertase gene (*TaCwi-A1*) and TKW, useful to improve grain yield. Li et al. [44] showed that expression of the cell wall invertase genes significantly improved shoot growth, grain yield and starch content in transgenic maize plants, and specifically increased both grain size and grain number.

*QTKW.mgb-6B* and *QGL.mgb-6B.2* were co-located on the second QTL cluster on 6B, suggesting that TKW improvement could be due to GL increase. In the same region, Elouafi and Nachit [45] reported a QTL for TKW using a linkage mapping population. Notably, the positive additive effect of the five QTL co-locating in the QTL clusters on 6B chromosome, was provided by the parental line Anco Marzio, indicating that this durum cultivar has potentially useful alleles for grain yield improvement.

Two QTL for grain size (*QGW.mgb-4B*, *QAREA.mgb-4B*) were found to co-localize on 4B chromosome. No QTL for grain size has been previously mapped in this region, indicating that it could be considered a new QTL cluster associated to grain size. The positive additive effect was provided by the parental line Liberdur. Among the candidate genes identified within the 4B cluster, one was particularly noticeable, a Big grain 1 protein (*TRITD4Bv1G171270*), which has been annotated as a positive regulator of auxin response and transport, as well as a regulator of grain size. The rice orthologue gene was characterized well by Liu et al. [25], who showed how its activation significantly improved grain size. This protein, localized in the plasma membrane, is induced by auxin treatment and its expression in vascular tissues could improve plant productivity, the most significant change being observed for increased grain size and grain weight.

The QTL cluster located on 1B was reported for the first time in this study, as no previous QTL for GW and GL have been reported in this region. Nevertheless, the positive additive effect of the GW QTL negatively affected the GL, suggesting that linked and/or pleiotropic genes for grain size map in this region.

QTL clusters on 3A and 7B chromosomes included a QTL for AREA, which co-localized with a TKW and a GW QTL, respectively, confirming the positive and highly significant correlation among TKW and grain size.

Searching for candidate genes highlighted two more interesting regions on 3A and 5A chromosomes, coincident with a PH QTL and a GL QTL, respectively (Figure 2). Indeed, the candidate gene screening on 3A chromosome identified a serine carboxypepdidase, *TRITD3Av1G187020*, and a NADH-dependent glutamate synthase (*NADH-GOGAT*), *TRITD3Av1G177110*, confirming their involvement in grain yield [38,39,46]. Additionally, an interesting gene was found in the GL QTL region on 5A chromosome, *TRITD5Av1G037950*, encoding for an expansin protein, a paralogue of *TRITD2Av1G019110* gene, reported in the first 2A QTL cluster, which belong to a gene family previously found related to both grain size and yield [47,48].

## 4. Materials and Methods

### 4.1. Genetic Materials and Phenotypic Analysis

The mapping population used in this study consists of 133 recombinant inbred lines (RILs) developed from a cross between two durum wheat cultivars, Liberdur and Anco Marzio, differing for yield-related traits, by advancing random individual F_2_ plants to the F_7_ generation by the single seed descent procedure. The RILs population was evaluated in open-field conditions over three consecutive growing seasons (2016, 2017, 2018) at Valenzano (Bari, Italy). The experimental design was a complete randomized block design with three replicates with each experimental unit consisting of a 5 m^2^ plot. Sowing density was always 350 seeds m^2^. The field experiments were supplied with 45 kg/ha N and 115 Kg/ha P_2_O_5_ in pre-sowing and 85 kg/ha N in top dressing each year.

Heading time (HT) was recorded in each field trial as the number of days from 1 March to 50% ear-emergence, corresponding to stage 55 on the Zadoks et al. [49] scale. Plant height (PH) was measured at complete maturity of plants, and grain yield (YLD) per plot was measured after harvesting and expressed in t/ha. The morphometric grain related traits were determined by digital imaging analysis. For each replication of each line, 10 g of kernels were scanned using high-resolution scanner-based image analysis. The images were processed using the Image-Pro Plus 7.0 software (Media Cybernetics, USA). Grain length (GL), grain width (GW), grain area (AREA), and kernels number were measured. The kernels number was used to calculate the thousand kernel weight (TKW).

### 4.2. Single Nucleotide Polymorphism (SNP) Genotyping and Linkage Map Construction

Genomic DNA from each RIL and parental line (Liberdur and Anco Marzio) was diluted to 50 ng/μL and further analyzed with the wheat 90K iSelect array [19]. Genotyping was performed by TraitGenetics GmbH [50] following the manufacturer’s recommendations as described in Akhunov et al. [51]. The genotyping assays were carried out using the Illumina iScan reader and performed using GenomeStudio software version 2011.1 (Illumina, San Diego, CA, USA).

Chi-squared tests were used to determine the goodness-of-fit at *p* > 0.001 of segregation ratios to expected 1:1 ratio for each SNP. All markers with more than 10% missing data or segregating as presence/absence in the mapping population were excluded from further analysis. Linkage analysis between markers and determination of the linear order of loci was performed by QTL IciMapping 4.1 using the BIN and MAP functions [52]. Grouping was performed using the independence LOD parameter, with groups showing a LOD 5. The Kosambi mapping function was used to calculate map distances. SNPs data from the durum consensus map [22] were used as anchor loci and for assigning linkage groups to specific chromosomes. Linkage groups were named according to the wheat chromosome nomenclature followed by a number when two or more linkage groups were found for one chromosome.

### 4.3. Statistical Analysis and QTL Detection

Analysis of variance (ANOVA) was performed to test the significance of differences among the RILs and replications, using the MSTAT-C 2.0 software [53]. The effects of replicates and genotypes were considered in the model in each year. Combined analysis across three years was carried out for all grain yield components and grain size traits.

Genetic variance (σ^2^_G_), environmental variance (σ^2^_Ɛ_), and variance due to genotypic x year interaction (σ^2^_GY_) were obtained by using the combined analysis of variance. Broad-sense heritability (h^2^_B_) was estimated by the ratio σ^2^_G_ /σ^2^_P_, where σ^2^_P_, is phenotypic variance (σ^2^_P_ = σ^2^_G_ + σ^2^_GY_ + σ^2^_Ɛ_) as reported by Singh and Ceccarelli [54]. Pearson phenotypic correlation coefficients (r) were calculated for all the traits across the years [55]. Mean of parental lines and RIL population, range, coefficient of variation (C.V.), were calculated for each field trials and across years.

The phenotypic mean values of each field trial (2016, 2017 and 2018) and the mean across trials were used for QTL analysis. An inclusive composite interval mapping (ICIM) method was employed for QTL mapping using IciMapping 4.1 software [52]. A threshold P value of 0.001 (−log10(P) ≥ 3.0) was used for QTL detection, P value frequently used for many agronomical important quantitative traits [56,57,58]; suggestive QTL were considered at the sub-threshold 2.5 < −log10(P) < 3.0 when declared at least in one year. A QTL was considered stable when detected at −log10(P) ≥ 3.0 in at least two years [41,58,59,60]. The phenotypic variation (PVE = R^2^) and additive effect were estimated for each detected QTL. Positive or negative additive effect indicates the increasing or decreasing effect of the parental line Liberdur. QTL were named according to the rules of International Rules of Genetic Nomenclature [61]. The QTL name combined the traits evaluated (‘YLD’, ‘PH’, ‘HT’, ‘TKW’, ‘GL’, ‘GW’, and ‘AREA’) and the Research Department (Genetics and Plant Breeding Section, University of Bari, ‘mgb’) that carried out the experiments. Graphical representation of linkage groups and QTL was carried out using MapChart 2.2 software [62].

### 4.4. Statistical Analysis and QTL Detection

QTL intervals detected on genetic map were physically mapped on the durum wheat reference genome Svevo [63]. Left and right flanking markers of each confidence interval were first searched in the durum consensus map [21], and then projected on Svevo genome. When the marker on the RIL mapping population was not mapped on the consensus map, the closest one was chosen.

The projected flanking markers were searched and positioned on the durum reference genome, and the annotated genes within each interval were screened based on their confidence and functional annotation. Candidate genes potentially involved in yield related traits were further investigated by analyzing the relation of synteny with other *Triticeae* as well as an orthologous search in other grass species [64].

## 5. Conclusions

This study showed how yield improvement could be pursued considering yield components (TKW, GL, GW and AREA), as well as phenology-related traits (PH and HT). These yield sub-components showed higher inheritance than grain yield, allowing a more accurate and powerful stable QTL detection. Physical anchoring of these QTL on the reference durum wheat genome cv. Svevo, enabled the identification of candidate genes affecting the genetic grain yield network. The availability of SNP markers within candidate genes sequences might represent a breeding strategy based on functional markers, determining a more efficient grain yield genetic gain.

## Figures and Tables

**Table 1:** Mean squares from the combined analysis of variance of grain yield (YLD), plant height (PH), heading time (HT), thousand kernel weight (TKW), grain length (GL), grain width (GW), grain area (AREA) in the Liberdur x Anco Marzio recombinant inbred line (RIL) mapping population evaluated at Valenzano (Bari, Italy) for three years.

Source of Variation	df	YLD	PH	HT	TKW	GL	GW	AREA
Block	2	43.247	295.371	6.526	92.291	0.037	0.046	2.108
Year (Y)	2	404.805b **	11448.769 ***	44492.070 ***	14608.951 ***	3.238 **	12.673 ***	624.712 ***
Error	4	11.698	68.460	3.852	141.014	0.120	0.192	8.839
RIL (G)	132	1.754 ***	196.336 ***	341.415 ***	122.325 ***	0.883 ***	0.089 ***	10.604 ***
GxY	264	2.470 ***	40.368 ***	28.770 ***	22.868 ***	0.041 ***	0.025 ***	1.643 ***
Error	792	0.425	14.143	1.184	8.573	0.005	0.007	0.341

** and ***: significant differences at 0.01 *p* and 0.001 *p*, respectively.

**Table 2:** Means, ranges, standard deviation (SD), coefficient of variation (CV), and heritability (h2B), of grain yield (YLD), plant height (PH), heading time (HT), thousand kernel weight (TKW), grain length (GL), grain width (GW), grain area (AREA) in the Liberdur x Anco Marzio RIL mapping population evaluated at Valenzano (Bari, Italy) for three years.

Trait	Year	Liberdur	Anco Marzio	*p*-Value ^1^	RIL	Range	SD	CV (%)	h^2^_B_
YLD (t/ha)									
	2016	5.07	2.98	*	4.81	2.54–7.63	1.25	17.26	0.66
	2017	3.61	4.12	n.s.	3.99	2.89–5.36	0.46	11.75	0.39
	2018	6.16	5.60	n.s.	5.99	3.91–7.43	0.68	11.95	0.37
Across years		4.974	4.23	*	4.93	3.95–5.90	0.44	13.21	0.00
PH (cm)									
	2016	88	98	*	91	74–102	5	3.99	0.68
	2017	74	88	*	81	69–90	4	4.59	0.58
	2018	80	93	*	86	71–102	6	5.06	0.60
Across years		81	93	*	86	75–96	5	4.35	0.43
HT (days from 01 March)									
	2016	46	21	*	33	20–46	8	3.61	0.98
	2017	50	35	*	40	33–55	6	2.51	0.98
	2018	59	50	*	54	47–63	3	2.02	0.90
Across years		52	35	*	42	33–54	6	2.57	0.77
TKW (g)									
	2016	47.4	56.8	*	51.8	41.2–64.6	4.9	5.49	0.72
	2017	41.6	40.5	n.s.	40.2	33.3–48.9	3.8	5.25	0.70
	2018	39.5	47.0	*	43.2	33.0–57.6	4.5	8.72	0.52
Across years		42.8	48.1	*	45.0	36.9–54.8	3.7	6.49	0.45
GL (mm)									
	2016	7.3	7.4	n.s.	7.4	6.6–8.1	0.4	1.06	0.95
	2017	7.5	7.1	*	7.2	6.5–7.9	0.3	0.89	0.96
	2018	7.0	7.5	*	7.2	6.5–7.9	0.3	1.11	0.94
Across years		7.3	7.3	n.s.	7.3	6.6–7.9	0.3	1.01	0.85
GW (mm)									
	2016	3.4	3.8	*	3.4	3.1–3.8	0.1	2.02	0.76
	2017	3.1	2.8	n.s.	3.1	2.9–3.3	0.1	1.89	0.74
	2018	3.1	3.2	*	3.2	2.9–3.5	0.1	3.66	0.46
Across years		3.2	3.3	n.s.	3.2	3.0–3.5	0.1	2.63	0.35
AREA (mm^2^)									
	2016	19.4	21.9	*	19.7	16.6–23.5	1.5	2.60	0.88
	2017	18.1	17.3	*	17.5	15.3–19.7	1.0	2.31	0.86
	2018	16.9	19.1	*	18.0	15.1–21.7	1.2	4.43	0.65
Across years		18.2	19.4	*	18.4	15.7–21.6	1.1	3.17	0.56

^1^ * significant difference at 0.05 *p* value between parental lines based on *t*-test; n.s. = not significant.

**Table 3:** Pearson correlation coefficients (*r*) among grain yield (YLD), plant height (PH), heading time (HT), thousand kernel weight (TKW), grain length (GL), grain width (GW), grain area (AREA) in the Liberdur x Anco Marzio RIL mapping population evaluated for three years (2016, 2017 and 2018) and across year.

Year	Trait	YLD	PH	HT	TKW	GL	GW
2016	PH	−0.07					
	HT	0.85 ***	−0.17 *				
	TKW	−0.41 ***	0.01	−0.48 ***			
	GL	−0.23 **	−0.15	−0.29 ***	0.76 ***		
	GW	−0.65 ***	0.14	−0.70 ***	0.83 ***	0.37 ***	
	AREA	−0.51 ***	−0.02	−0.57 ***	0.956 ***	0.85 ***	0.80 ***
2017	PH	0.32 ***					
	HT	−0.36 **	−0.21 *				
	TKW	0.31 ***	0.10	−0.21 *			
	GL	−0.08	−0.11	0.26 **	0.56 ***		
	GW	0.26 **	0.13	−0.14	0.83 ***	0.19 *	
	AREA	0.08	−0.07	0.13	0.87 ***	0.85 ***	0.67 ***
2018	PH	0.40 ***					
	HT	−0.24 **	0.04				
	TKW	0.22 *	0.15	0.02			
	GL	0.01	−0.13	0.05	0.62 ***		
	GW	0.21 *	0.19 *	−0.01	0.90 ***	0.28 ***	
	AREA	0.14	0.04	0.02	0.94 ***	0.83 ***	0.77 ***
Across years	PH	0.16					
	HT	0.55 ***	−0.11				
	TKW	0.01	0.09	−0.24 **			
	GL	0.03	−0.17	0.01	0.70 ***		
	GW	−0.10	0.19 *	−0.36 ***	0.83 ***	0.23 **	
	AREA	−0.04	−0.02	−0.19 *	0.95 ***	0.86 ***	0.70 ***

*, ** and ***: significant differences at 0.05, 0.01 and 0.001 P, respectively.

**Table 4:** Distribution of single nucleotide polymorphism (SNP) markers across the 14 durum wheat chromosomes in the genetic linkage map Liberdur x Anco Marzio RILs mapping population.

Chromosome	Linkage Groups (n°)	Total Markers (n°)	Map Length (cM)	Marker Density (SNP/cM)
1A	2	230	137.0	1.7
1B	1	587	154.8	3.8
2A	1	355	195.1	1.8
2B	2	417	165.8	2.5
3A	2	300	141.4	2.1
3B	1	551	187.0	2.9
4A	3	170	139.7	1.2
4B	1	273	147.2	1.9
5A	2	316	175.5	1.8
5B	2	226	73.3	3.1
6A	1	264	159.4	1.7
6B	1	490	155.0	3.2
7A	1	450	197.2	2.3
7B	1	505	179.7	2.8
Genome A	12	2085	1145.3	1.8
Genome B	9	3049	1062.7	2.9
Total	21	5134	2207.9	2.3

**Table 5:** Quantitative trait loci (QTL) for grain yield (YLD), plant height (PH), heading time (HT), thousand kernel weight (TKW), grain length (GL), grain width (GW), grain area (AREA) in the Liberdur x Anco Marzio RILs mapping population evaluated at Valenzano (Bari, Italy) for three years.

Trait	QTL	Linkage Group	Left Marker	Right Marker	Interval (cM)	Years									Across Years		
						2016			2017			2018					
						P	Add	R^2^ (%)	P	Add	R^2^ (%)	P	Add	R^2^ (%)	P	Add	R^2^ (%)
YLD	*QYLD.mgb-2A*	2A	IWB70473	IWB70098	34.4–35.6	36.5	1.04	67.9	-			-					
	*QYLD.mgb-7B*	7B	IWB16239	IWB35358	41.5–46.7	-			-			3.7	−0.24	11.9			
HT	*QHT.mgb-2A*	2A	IWB70473	IWB70098	34.4–35.6	69.7	8.66	85.7	50.4	5.78	75.7	44.6	3.00	74.3	62.4	5.79	82.9
	*QHT.mgb-7A*	7A	IWB7484	IWB10719	55.7–64.8	-			3.8	1.05	2.5	*2.8*	0.53	2.4	3.1	0.82	1.7
PH	*QPH.mgb-2B*	2B_2	IWB42187	IWB51104	14.0–17.1	-			-			4.3	−1.87	10.3	5.8	−1.56	11.9
	*QPH.mgb-3A*	3A_1	IWB71668	IWB6774	57.3–64.0	6.2	2.09	12.8	-			3.9	1.75	9.2	5.3	1.48	10.9
	*QPH.mgb-6B*	6B	IWB42532	IWB7935	4.3–6.8	7.9	−2.45	17.5	-			3.2	−1.62	7.8	5.8	−1.59	12.5
	*QPH.mgb-7A*	7A	IWB43996	IWB4178	88.3–94.7	-			-			3.1	−1.62	10.0	4.1	−1.33	8.8
TKW	*QTKW.mgb-1A*	1A	IWB6172	IWB21573	14.4–20.6	*2.8*	−0.82	2.9	-			3.0	−1.18	6.8	4.1	−0.83	5.1
	*QTKW.mgb-2A.1*	2A	IWB70098	IWB70375	35.6–36.4	19.6	−2.52	27.1	3.5	−0.81	4.7	-			5.9	−1.01	7.4
	*QTKW.mgb-2A.2*	2A	IWB62891	IWB26699	87.5–89.6	19.3	2.51	26.2	19.4	2.26	35.8	12.2	2.61	32.6	23.0	2.37	40.3
	*QTKW.mgb-3A*	3A_1	IWB5723	IWB25484	20.8–21.6	5.3	1.19	5.7	-			-			3.0	0.71	3.6
	*QTKW.mgb-6B*	6B	IWB26622	IWB73386	87.9–88.3	7.5	−1.42	8.5	-			-			7.1	−1.11	9.1
GL	*QGL.mgb-1B*	1B	IWB67970	IWB6409	45.1–46.7	5.8	−0.09	6.4	-			8.4	−0.09	9.2	-		
	*QGL.mgb-2A.1*	2A	IWB70098	IWB70375	35.6–36.4	12.7	−0.13	15.1	-			-			-		
	*QGL.mgb-2A.2*	2A	IWB62891	IWB26699	87.5–89.6	25.5	0.21	39.1	28.2	0.24	53.8	29.8	0.21	48.8	31.6	0.24	51.2
	*QGL.mgb-5A*	5A_1	IWA4765	IWB36812	36.8–47.3	3.8	−0.07	4.8	-			4.0	−0.06	4.8	*2.8*	−0.06	2.8
	*QGL.mgb-6B.1*	6B	IWB27151	IWA4610	3.9–4.3	*2.6*	−0.10	2.6	-			4.5	−0.06	4.5	-		
	*QGL.mgb-6B.2*	6B	IWB19680	IWB70152	89.8–99.1	-			-			7.8	−0.09	8.3	3.9	−0.06	3.7
GW	*QGW.mgb-1B*	1B	IWB34334	IWB51878	47.1–48.4	8.1	0.10	9.3	3.9	0.10	8.4	4.8	0.10	12.7	8.8	0.04	17.0
	*QGW.mgb-2A.1*	2A	IWB70098	IWB70375	35.6–36.4	26.1	−0.10	42.0	-			-			4.6	−0.03	8.4
	*QGW.mgb-2A.2*	2A	IWB62891	IWB25623	87.5–93.5	-			7.0	0.10	16.3	3.5	0.10	9.2	6.6	0.04	12.1
	*QGW.mgb-4B*	4B	IWB26975	IWB17082	66.0–70.4	-			-			3.0	0.10	8.0	-		
	*QGW.mgb-6B*	6B	IWA1254	IWB42821	2.0–2.4	3.9	−0.10	4.1	-			-			-		
	*QGW.mgb-7B*	7B	IWB70613	IWB69446	107.9–108.3	-			4.5	0.1	9.8	-			*2.7*	0.1	4.7
AREA	*QAREA.mgb-2A.1*	2A	IWB70098	IWB70375	35.6–36.4	26.8	−0.90	35.4	-			-			6.3	−0.28	6.6
	*QAREA.mgb-2A.2*	2A	IWB62891	IWB26699	87.5–89.6	21.6	0.77	25.8	26.6	0.70	47.8	19.0	0.74	43.8	28.1	0.75	44.9
	*QAREA.mgb-3A*	3A_1	IWB5723	IWB25484	20.8–21.6	3.1	0.26	2.8	-			-			-		
	*QAREA.mgb-4B*	4B	IWB38381	IWB26975	64.7–66.0	-			4.8	0.24	5.6	3.2	0.26	5.4	-		
	*QAREA.mgb-7B*	7B	IWB11767	IWB68453	89.9–99.1	-			4.7	0.27	6.6	-			*2.6*	0.19	2.8

Percentage of the phenotypic variance(R^2^) and additive effect (Add) are reported in the year and across years when the QTL or suggestive QTL was detected. Suggestive QTL significant at 2.5 ≤ −log10(P) ≤ 3.0 are reported in italic. - Not significant.

**Table 6:** Physical position and candidate genes within the QTL cluster detected on Liberdur x Anco Marzio genetic map evaluated for grain yield (YLD), plant height (PH), heading time (HT), thousand kernel weight (TKW), grain length (GL), grain width (GW), grain area (AREA).

QTL Cluster	Trait Associated	Chromosome	Genetic Position on LA Map (cM)	Physical Position		Gene ID	Candidate Function
				Start (Mbp)	End (Mbp)		
1	GL, GW	1B	45.1–48.4	348.3	361.8	*TRITD1Bv1G120210*	RING U-box superfamily protein
						*TRITD1Bv1G119290*	Cytochrome P450 family protein
						*TRITD1Bv1G118820*	Auxin-responsive family protein
						*TRITD1Bv1G118960*	Serine carboxypeptidase
						*TRITD1Bv1G119070*	Cytochrome P450 family protein
2	HT, YLD, TKW, GL, GL, AREA	2A	34.4–36.4	36.3	40.2	*TRITD2Av1G019110*	Expansin protein
						*TRITD2Av1G019200*	RING U-box superfamily protein
						*TRITD2Av1G019220*	E3 ubiquitin-protein ligase
						*TRITD2Av1G019250*	Pseudo-response regulator *(Ppd-A1)*
3	TKW, GL, GW, AREA	2A	87.5–93.5	510.0	560.6	*TRITD2Av1G183880*	Cellulose synthase family protein
						*TRITD2Av1G184660*	Thioredoxin
						*TRITD2Av1G187690*	Mitogen-activated protein kinase
						*TRITD2Av1G189400*	Auxin response factor (*ARF1*)
						*TRITD2Av1G190210*	Thioredoxin family protein
						*TRITD2Av1G192750*	Serine carboxypeptidase
4	TKW, AREA	3A	20.8–21.6	22.5	22.9	*TRITD3Av1G011970*	Kinase family protein
						*TRITD3Av1G012140*	Receptor-like kinase
						*TRITD3Av1G012070*	Flavin-containing monooxygenase
5	GW, AREA	4B	64.7–70.4	567.5	598.6	*TRITD4Bv1G171270*	Protein Big grain 1
						*TRITD4Bv1G172000*	E3 ubiquitin-protein ligase
						*TRITD4Bv1G175480*	Auxin signaling
						*TRITD4Bv1G175840*	RING U-box superfamily protein
6	PH, GL, GW	6B	2.0–6.8	10.8	24.7	*TRITD6Bv1G003650*	Ubiquitin family protein
						*TRITD6Bv1G005100*	Cytochrome P450
						*TRITD6Bv1G005370*	Acid β-fructofuranosidase
						*TRITD6Bv1G005450*	Acid β-fructofuranosidase
7	TKW, GL	6B	87.1–99.1	556.1	613.2	*TRITD6Bv1G185080*	E3 ubiquitin-protein ligase
						*TRITD6Bv1G189120*	Thioredoxin
8	GW, AREA	7B	89.9–108.3	543.8	579.0	*TRITD7Bv1G171980*	E3 ubiquitin-protein ligase
						*TRITD7Bv1G173200*	Auxin response factor
						*TRITD7Bv1G174660*	Cytochrome P450
						*TRITD7Bv1G174890*	E3 ubiquitin-protein ligase
						*TRITD7Bv1G180500*	RING U-box superfamily protein

## Data Availability

Data is contained within the article or supplementary material.

## Supplementary Materials

The following are available online at https://www.mdpi.com/2223-7747/10/2/312/s1, Table S1: Analysis of variance of grain yield (YLD), plant height (PH), heading time (HT), thousand kernel weight (TKW), grain length (GL), grain width (GW), grain area (AREA) in the Liberdur x Anco Marzio RILs mapping population evaluated at Valenzano (Bari, Italy) for three years.
