# Supplementary material for: Candidate Genes and Quantitative Trait Loci for Grain Yield and Seed Size in Durum Wheat
Source: Plants (Basel). 2021 Feb 5;10(2):312. doi: 10.3390/plants10020312 (PMC7916090; doi:10.3390/plants10020312)
Supplement: Supplementary file 1 [file plants-10-00312-s001.pdf]

Table S1. Analysis of variance of grain yield (YLD), plant height (PH), heading time (HT), thousand kernel weight (TKW), grain length (GL), grain width (GW), grain area (AREA) in the Liberdur x Anco Marzio RIL mapping population evaluated at Valenzano (Bari, Italy) for three years.

Year	Source of variation	df	YLD	PH	HT	TKW	GL	GW	AREA
2016	Block	2	52.110***	8.674	3.619	94.895***	0.131***	0.195***	8.247***
	RIL	132	4.720***	96.489***	239.341***	70.877***	0.364***	0.064***	6.416***
	Error	264	0.687	13.310	1.421	8.089	0.006	0.006	0.267
2017	BLock	2	7.422***	375.387***	2.279	14.845*	0.054***	0.001	0.297
	RIL	132	0.639***	71.095***	122.914***	36.252***	0.307***	0.028***	3.209***
	Error	264	0.220	13.704	1.033	4.446	0.004	0.003	0.164
2018	Block	2	7.560***	63.550*	8.021*	264.174***	0.103***	0.236***	11.557***
	RIL	132	1.408***	107.644***	34.928***	59.814***	0.291***	0.046***	4.166***
	Error	264	0.514	19.352	1.185	14.168	0.006	0.013	0.634

*, ** and ***: significant differences at 0.05P, 0.01P and 0.001P, respectively.
